# Supplementary material for: Tetraspanin 8 Subfamily Members Regulate Substrate-Specificity of a Disintegrin and Metalloprotease 17
Source: Cells. 2022 Aug 29;11(17):2683. doi: 10.3390/cells11172683 (PMC9454446; doi:10.3390/cells11172683)
Supplement: Supplementary file 1 [file cells-11-02683-s001.zip › cells-1850761-supplementary.pdf]

Supplementary data to:

Tetraspanin 8 Subfamily Members Regulate Substrate-Specificity of a Disintegrin and Metalloprotease 17

Miryam Müller, Claire Saunders, Anke Senftleben, Johannes P. W. Heidbuechel, Birgit Halwachs, Julia Bolik, Nina Hedemann, Christian Röder, Dirk Bauerschlag, Stefan Rose-Johnand Dirk Schmidt-Arras

Supplementary Experimental Procedures

Supplementary Table S1 - siRNAs used in this study

Target	target species	Sequence	Distributed by	Product nr.
siCtrl	human/mouse		Dharmacon/Horizon	D-001810-10-05 OnTarget Plus Non-targeting
siADAM17-1	human		Dharmacon/Horizon	L-010219-00-0005 OnTarget Plus

Supplementary Table S2 – primary antibodies used in this study

Target	Host species	Dilution	Distibuted by
**Western Blot**			
anti-CO-029	mouse	1:250	kind gift of Margot Zöller, Heidelberg University
anti-CD9 (sc-13118)	mouse	1:1,000	Santa-Cruz Biotechnology, Heidelberg, Germany
anti-CD81 (sc-7637)	mouse	1:1,000	Santa-Cruz Biotechnology, Heidelberg, Germany
anti-ADAM10 (ab39162)	rabbit	1:2,000	abcam, Cambridge, UK
anti-ADAM17 (ab124695)	rabbit	1:1,000	Chemicon®, Merck, Darmstadt, Germany
P-p38 (Thr180/Tyr182) (clone D3F9) (#4511)	rabbit	1:1,000	Cell signaling/NEB, Frankfurt a. M., Germany
p38 (clone D13E1) (#8690)	rabbit	1:1,000	Cell signaling/NEB, Frankfurt a. M., Germany
β-catenin (clone D10A8) (#8480)	rabbit	1:1,000	Cell signaling/NEB, Frankfurt a. M., Germany

glutamine synthetase (GS, 610517)	mouse	1:5,000	BD Biosciences, Heidelberg, Germany
P-STAT3 (clone D3A7) (#9145)	rabbit	1:2,000	Cell signaling/NEB, Frankfurt a. M., Germany
STAT3(clone 124H6) (#9139)	mouse	1:1,000	Cell signaling/NEB, Frankfurt a. M., Germany
P-Erk1/2 (Thr202/Tyr204) (clone D13.14.4E) (#4370)	rabbit	1:1,000	Cell signaling/NEB, Frankfurt a. M., Germany
Erk1/2 (clone 137F5) (#4695)	mouse	1:1,000	Cell signaling/NEB, Frankfurt a. M., Germany
β-actin (A2066)	rabbit	1:10,000	Sigma-Aldrich, Steinheim, Germany
anti-TNFα (840120)	goat	1:1,000	R&D Systems, Wiesbaden, Germany
**Immunofluorescence**			
anti-CO-029	mouse	1:25	kind gift of Margot Zöller, Heidelberg University
anti-CD9 (sc-13118)	mouse	1:100	Santa-Cruz Biotechnology, Heidelberg, Germany
anti-CD81 (sc-7637)	mouse	1:100	Santa-Cruz Biotechnology, Heidelberg, Germany
anti-TNFα (840120)	goat	1:180	R&D Systems, Wiesbaden, Germany
anti-TNFα (3707)	rabbit	1:100	Cell signaling/NEB, Frankfurt a. M., Germany
**Flow Cytometry**			
anti-huCD9-APC (clone HI9a) (#312107)	mouse	1:25	BioLegend
anti-huCD81-PerCP/Cy5.5 (clone 5A6) (#349507)	mouse	1:25	BioLegend
anti-huTspan8-PE (clone TAL69) (#363703)	mouse	1:25	BioLegend

Supplementary Table S3 – secondary antibodies used in this study

Target	Host species	Dilution	Distibuted by
**Immunofluorescence**			
anti-goat AF594 (A21209)	donkey	1:200	Thermo Fisher Scientific, Darmstadt, Germany
anti-mouse AF488 (A21202)	donkey	1:200	Thermo Fisher Scientific, Darmstadt, Germany
**STED microscopy**			

anti-rabbit AF594 (21207)	donkey	1:200	Thermo Fisher Scientific, Darmstadt, Germany
anti-mouse StarRed	goat	1:200	abberior Instruments GmbH, Göttingen, Germany

Supplementary Figures

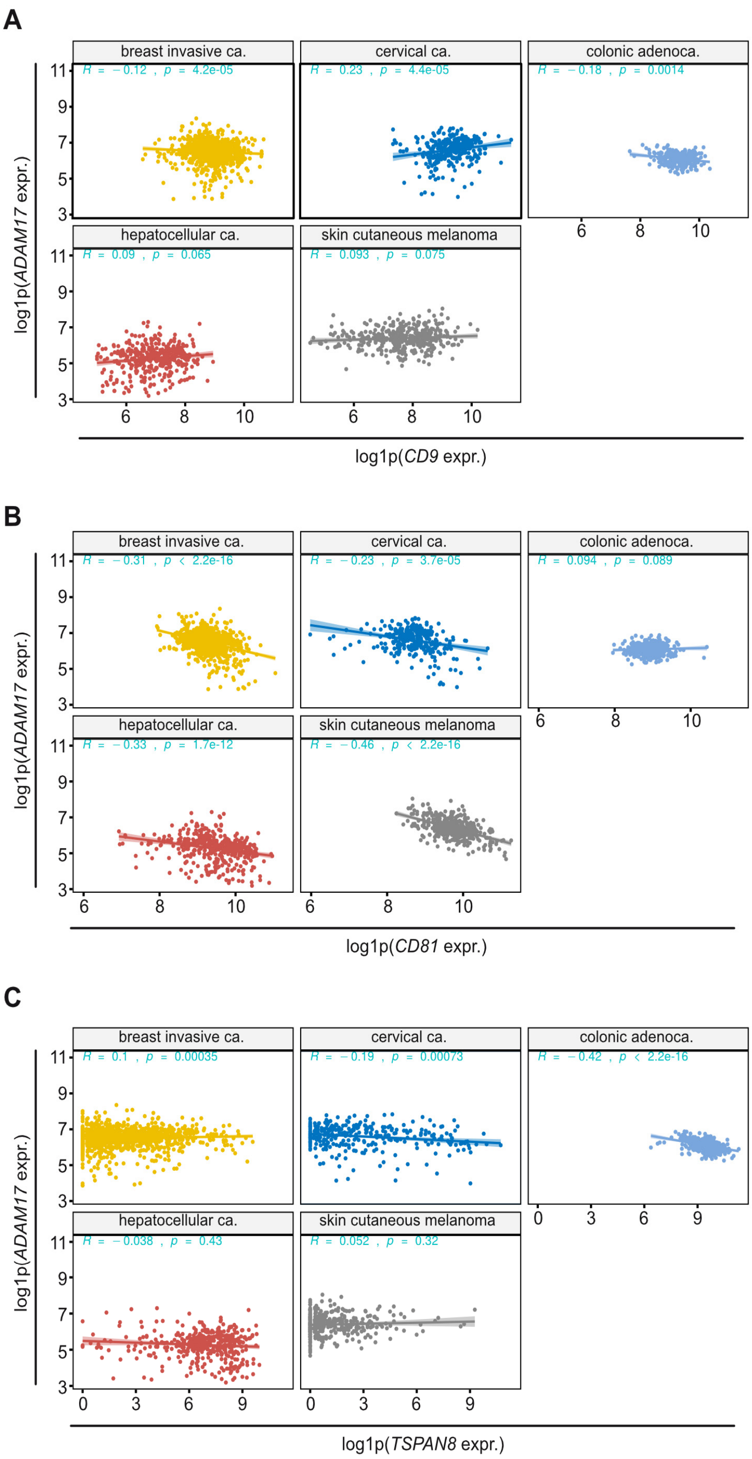

Supplementary Figure S1: Correlation of Tspan8 subgroup member expression with ADAM17 expression within different tumour subsets. A-C. Gene expression data of the indicated genes encoding different tetraspanins and ADAM17 were retrieved from The Cancer Genome Atlas (TCGA) database and subjected to correlation analysis.

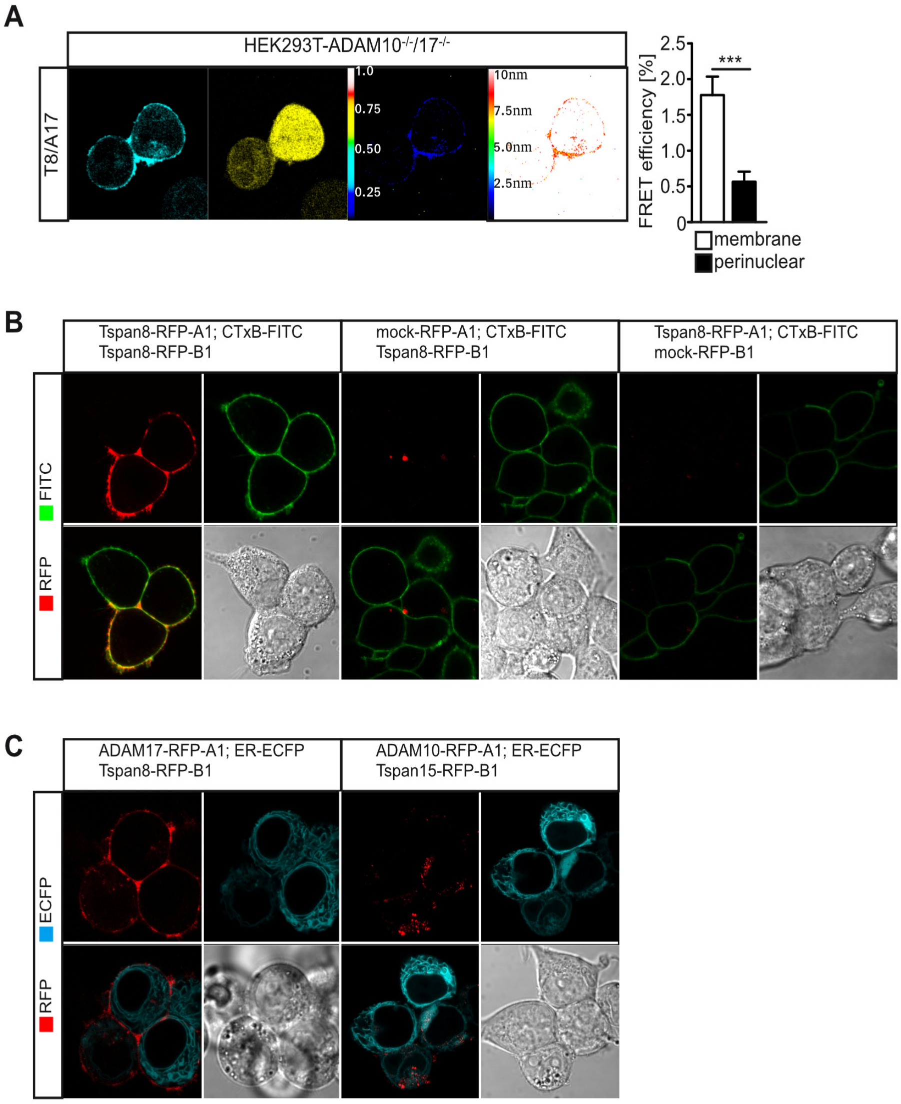

Supplementary Figure S2: Tspan8 associates with ADAM17 at the plasma membrane. A. HEK293T cells deficient for ADAM10 and ADAM17 were transfected with expression constructs coding for Tspan8-CyPet and ADAM17-YPet fusion and Tspan8-ADAM17 association was measured by confocal microscopy and fluorescence resonance energy transfer (FRET). **B.** HEK293T cells were transfected coding for Tspan8 fused to individual non-fluorescent RFP monomers as indicated and incubated with FITC-labeled cholera toxin B (CtxB) before confocal microscopy analysis. Tspan8 multimerisation is indicated by red fluorescence. **C.** HEK293T cells were co-transfected with KDEL-fused ECFP (ER-ECFP) and expression constructs coding for either Tspan8 or 15 and ADAM17 or 10, respectively, fused to the indicated non-fluorescent RFP monomer. Cells

were analysed by confocal microscopy. Data are represented as mean±s.e.m., n=3 independent experiments (A), ***$P<0.001$, Kruskal-Wallis with Dunn's post-hoc test (A).

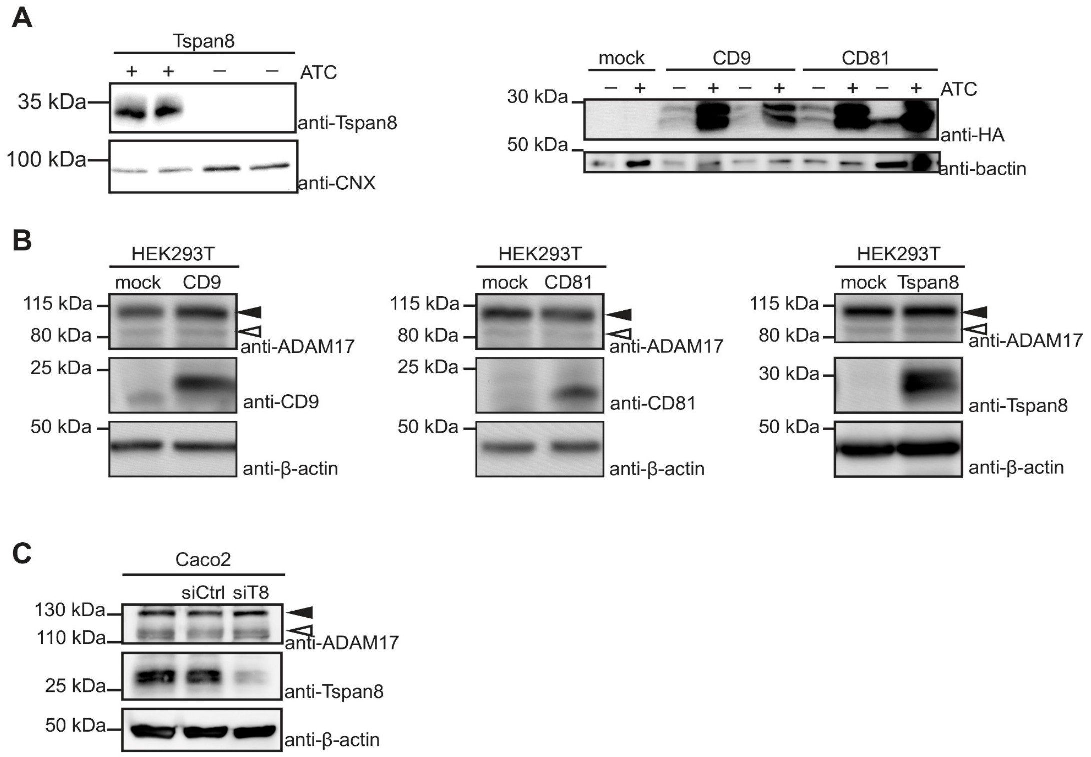

Supplementary Figure S3: Generation of stable HEK293T cells with inducible Tspan-expression and siRNA-mediated Tspan8 down-regulation in Caco2 colon cancer cells A. HEK293T cells stably transfected with tetracycline-inducible expression constructs coding for the indicated tetraspanin were induced with 100 ng/mL anhydrotetracyclin (ATC) and subjected to cell lysis, SDS-PAGE and immunoblotting using the indicated antibodies. **B.** Overexpression of Tspan8 family members does not alter ADAM17 maturation as assessed by immunoblotting using the indicated antibodies. **C.** siRNA-mediated downregulation of Tspan8 does not affect ADAM17 maturation in the Caco2 colon carcinoma cell line ass assessed by immunoblotting using the indicated antibodies.

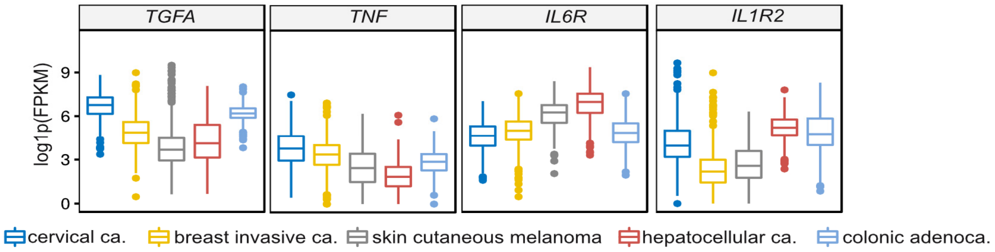

Supplementary Figure S4: Expression of selected ADAM17 substrates in tumour subsets. RNAseq gene expression data of the indicated genes encoding ADAM17 substrates were retrieved from The Cancer Genome Atlas (TCGA) database and classified by the indicated tumour entities.

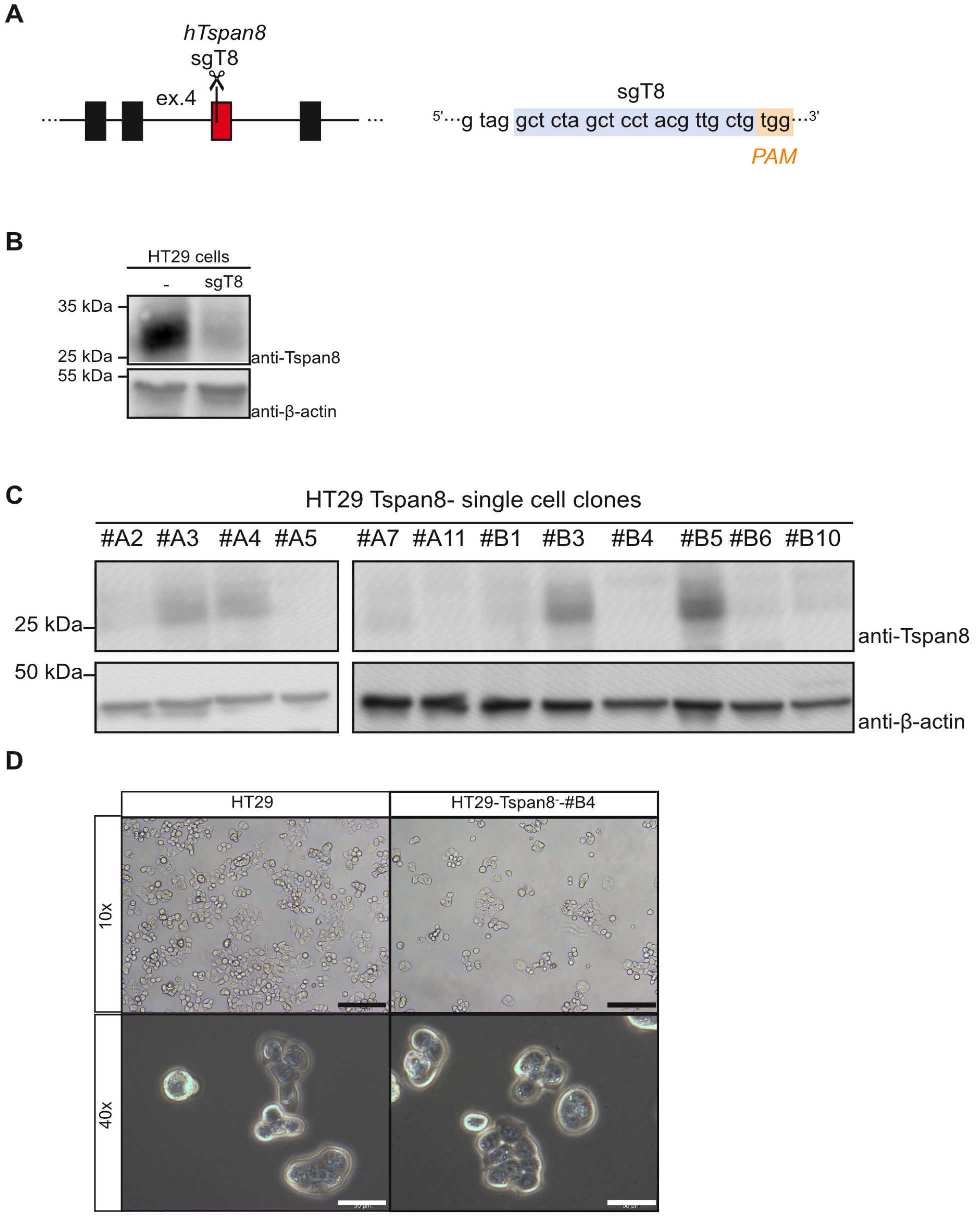

Supplementary Figure S5: Generation of HT29 cells with CRISPR/Cas9-mediated Tspan8-deficiency. A. Design of sgRNA (sgT8) targeting exon 4 of the human *Tspan8* gene. **B.** Absence of Tspan8 expression in CRISPR-tageted HT29 cells was verified by SDS-PAGE and immunoblotting using the indicated antibodies. **C.** Single cell clone analysis of HT29 cells with CRISPR-mediated knockout of *Tspan8*. **D.** Morphology of Tspan8-deficient HT29 single cell clone is not altered as compared to parental HT29 cells. Scale bar indicates 200 µm (upper panel) or 50 µm (lower panel).

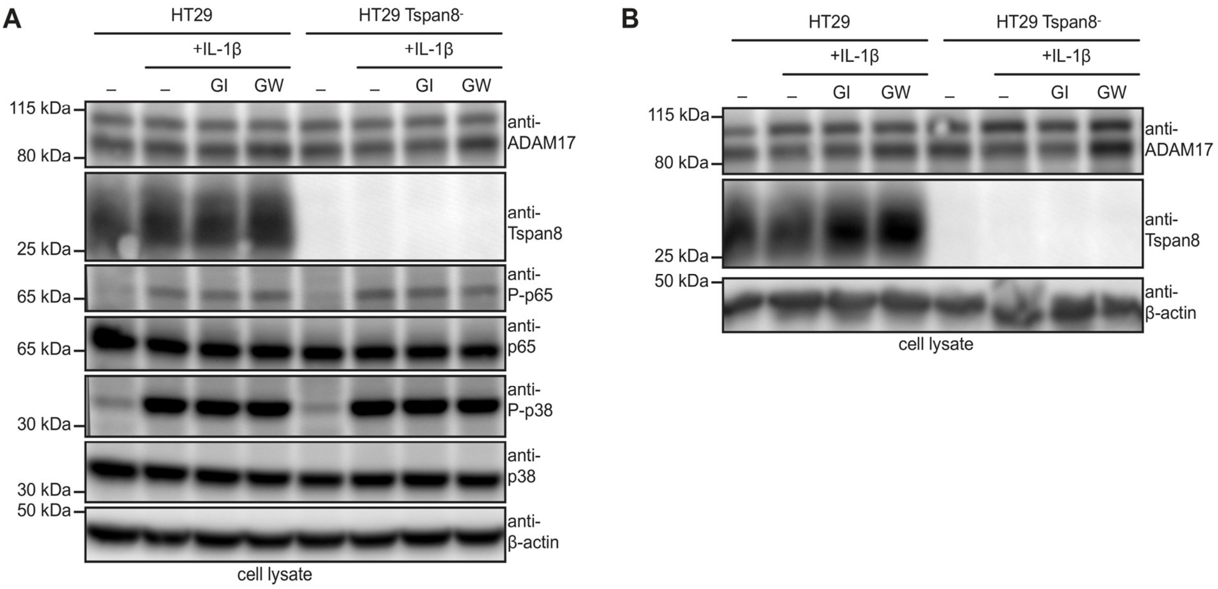

Supplementary Figure S6: Tspan8 does not influence ADAM17 maturation or IL-1β downstream signalling. A. CRISPR-mediated Tspan8-deficiency in the HT29 colon carcinoma cell line does neither alter ADAM17 maturation nor does it affect downstream signalling of IL-1β as assessed by immunoblotting using the indicated antibodies. Cells were pre-incubated with 3 μM GI254023X or 3 μM GW280264X for 30 min, stimulated for 15 min with 20 ng/mL IL-1β, subsequently lysed and subjected to SDS-PAGE and immunoblotting. **B.** The indicated cell lines were stimulated for 4 hours with 20 ng/mL IL-1β, subsequently lysed and subjected to SDS-PAGE and immunoblotting using the indicated antibodies.
